# Frequency of deep vein thrombosis at admission for acute stroke and associated factors: a cross-sectional study

**DOI:** 10.1186/s12959-021-00315-5

**Published:** 2021-09-06

**Authors:** Takahisa Mori, Kazuhiro Yoshioka, Yuhei Tanno

**Affiliations:** 1 Department of Stroke Treatment, Shonan Kamakura General Hospital, Okamoto 1370-1, 247-8533 Kamakura City, Kanagawa Japan; 2 Department of Neurology, Nakatsugawa Municipal General Hospital, Komaba 1522-1, Gifu 508-8502 Nakatsugawa City, Japan

**Keywords:** Deep vein thrombosis, D-dimer, Frequency, Intermittent pneumatic compression, Stroke, Ultrasonography

## Abstract

**Background:**

Intermittent pneumatic compression (IPC) is commonly used to prevent deep vein thrombosis (DVT) during hospitalization in patients with acute stroke. However, if DVT exists at admission, IPC of the legs with DVT may cause migration of the thrombi, resulting in pulmonary emboli. Whole-leg ultrasonography (wl-US) is a practical tool to detect DVT; however, wl-US is not always performed at admission in all stroke patients. This retrospective cross-sectional study aimed to investigate DVT frequency and identify significant factors indicating the presence of DVT at admission for acute stroke.

**Methods:**

We included patients admitted within 24 h of stroke onset between 2017 and 2019. Patients who did not undergo blood tests for D-dimer or wl-US within 72 h of arrival were excluded. We collected patient data on age; sex; anthropometric variables; presence of DVT on wl-US; and biomarkers such as D-dimer, high-sensitivity C-reactive protein (hs-CRP), and lipids.

**Results:**

Of 1129 acute stroke patients, 917 met our inclusion criteria. DVT was detected in 161 patients (17.6 %). Patients with DVT were older; were more likely to be female; had lower body weight; had higher D-dimer and hs-CRP levels; had lower albumin, hemoglobin, and triglyceride levels; and had higher National Institutes of Health Stroke Scale and pre-stroke modified Rankin scale scores than patients without DVT (*n* = 756). In addition, multiple logistic regression analysis showed that sex (female) and D-dimer levels (≥ 1.52 µg/mL) were independent significant factors for the presence of DVT. Among 161 patients with DVT, 78 (48.4 %) had both these significant factors. Among 756 patients without DVT, 602 (79.6 %) had no or one significant factor. The odds ratio of the presence of DVT in patients with both significant factors was 6.29, using patients without any significant factors as the group for comparison.

**Conclusions:**

The frequency of DVT is high in acute stroke patients at admission. Female sex and a high D-dimer level were independent significant factors for the presence of DVT. Therefore, in patients with these two significant factors at admission, IPC should be avoided or wl-US should be performed before IPC.

**Supplementary Information:**

The online version contains supplementary material available at 10.1186/s12959-021-00315-5.

## Background

Venous thromboembolism (VT) is a common cause of death and morbidity in patients with acute stroke during hospitalization [1]. Anticoagulants reduce the frequency of pulmonary emboli due to VT; however, this benefit is offset by an increase in the frequency of extracranial hemorrhage [2]. In addition, anticoagulants cannot be administered to patients with hemorrhagic stroke. Therefore, intermittent pneumatic compression (IPC) is commonly used to reduce the risk of deep vein thrombosis (DVT) during hospitalization [3]. A DVT frequency of 8.0 or 8.7 % at admission was reported from Polish institutions [4, 5]; however, the DVT frequency at admission has been unknown in Japan. If the DVT frequency is high in Japan and IPC of the legs with DVT is started in patients with DVT at the time of admission, IPC may cause migration or fragmentation of thrombi and lead to pulmonary embolism. During hospitalization, D-dimer levels are often elevated in patients with DVT [6]. Whole-leg ultrasonography (wl-US) is a practical tool to detect DVT in outpatients or inpatients [7]. wl-US or D-dimer measurement should always be performed for detecting DVT at admission for stroke; however, wl-US is not routinely performed at admission in many facilities. Therefore, a practical index to estimate the presence of DVT at stroke admission is necessary. Our retrospective cross-sectional study aimed to investigate DVT frequency at admission and identify significant factors specific to the presence of DVT at admission for acute stroke.

## Methods

To investigate DVT frequency at admission and identify related factors, we included patients admitted within 24 h of stroke onset between March 2017 and March 2019. We excluded patients whose plasma D-dimer level was not examined within 24 h of arrival or in whom whole-leg US was not performed within 72 h of arrival. We collected patient data on age, sex, anthropometric variables, and US findings of DVT. We evaluated biomarkers such as hemoglobin (Hb), serum albumin (Alb), high-sensitivity C-reactive protein (hs-CRP), glucose, HbA1c, total cholesterol, high-density lipoprotein cholesterol, triglycerides (TG), aspartate aminotransferase (AST), alanine aminotransferase (ALT), AST/ALT ratio, and plasma D-dimer. In addition, we evaluated the National Institutes of Health Stroke Scale (NIHSS) score [8] and pre-stroke modified Rankin scale (mRS) score at admission [9]. The low-density lipoprotein cholesterol concentration was calculated using the Friedewald formula: low-density lipoprotein cholesterol = total cholesterol – high-density lipoprotein cholesterol – TG/5. D-dimer levels were measured using latex turbidimetric immunoassay (LIAS AUTO D-Dimer NEO, Sysmex Co., Hyogo, Japan) [10]. DVT was diagnosed according to the findings of wl-US (Xario, Canon Medical Systems Co., Tochigi, Japan), performed by trained radiologists [11]. DVT was diagnosed based on the following US findings: presence of a non-compressible segment or flow impairment on color Doppler imaging [12]. Compression was performed at 2 cm intervals.

### Statistical analysis

Non-normally distributed continuous variables are expressed as medians and interquartile ranges. We compared all possible pairs of variables with significant differences between patients with and without DVT. A dummy variable was used to represent categorical data, such as data on sex, and Spearman rank correlation coefficient (*r*_s_) was calculated to measure the strength of the relationships. We defined 0 ≤ |*r*_s_| < 0.1 as no correlation, 0.1 ≤ |*r*_s_| < 0.4 as a weak correlation, 0.4 ≤ |*r*_s_| < 0.6 as a moderate correlation, and 0.6 ≤ |*r*_s_| as a strong correlation. Multicollinearity was defined as the presence of a moderate or strong correlation between variables. When variables were moderately or strongly correlated with one another, we adopted the variable with a larger chi-squared value. After excluding variables with multicollinearity, we conducted a multiple logistic regression analysis to identify independent variables indicating the presence of DVT. We estimated the threshold values of independent variables indicating the presence of DVT using area under the curve values derived from the receiver operating characteristic curves of the logistic regression model. Statistical significance was set at a *P*-value < 0.05. We used JMP software (version 16.0; SAS Institute, Cary, NC, USA) for all statistical analyses.

## Results

During the study period, 1129 acute stroke patients were admitted, and 917 met our inclusion criteria. Patient characteristics are summarized in Table 1. DVT was detected in 161 (17.6 %) of 917 patients at admission. Patients with DVT (*n* = 161) were older; were more likely to be female; had lower body weight; had higher plasma D-dimer and hs-CRP levels; had lower Hb, serum Alb, TG, and ALT levels; had a higher AST/ALT ratio; and had higher NIHSS and pre-stroke mRS scores than patients without DVT (*n* = 756) (Table 2). After excluding variables with significant differences between the two groups and variables with multicollinearity (Additional file 1), multiple logistic regression analysis showed that sex and D-dimer levels were independent variables for the presence of DVT (Table 3). Receiver operating characteristic curves demonstrated that female sex and a D-dimer level of ≥ 1.52 µg/mL were independent factors for the presence of DVT at the time of stroke admission (Table 4). Among 161 patients with DVT, 78 (48.4 %) had both these significant factors. Among 756 patients without DVT, 602 (79.6 %) had no or one significant factor. The odds of the presence of DVT was 0.506 in patients with both significant factors, 0.182 in patients with one significant factor, and 0.081 in patients without any significant factors. On using patients without any significant factors as the group for comparison, the odds ratio of the presence of DVT was 6.29 in patients with both significant factors (Table 5).

**Table 1:** Patient characteristics

Variables	Values	
N	917	
DVT, n (%)	161 (17.6 %)	
Ischemic stroke, n (%)	734 (80.0 %)	
Female sex, n (%)	449 (49.0 %)	
Age, years	80 (71, 86)	
BMI, kg/m^2^	22.0 (19.5, 24.4)	
BW, kg	55 (47, 65)	
Hb, g/dL	13.3 (12, 14.6)	
Plt, /µL	20.8 (17.1, 25.5)	
Alb, mg/dL	3.9 (3.6, 4.2)	
AST, U/L	23 (19, 29)	
ALT, U/L	16 (12, 23)	
AST/ALT ratio	1.4 (1.1, 1.8)	
Glucose, mg/dL	122 (105, 151)	
HbA1c, % (NGSP)	5.8 (5.5, 6.4)	
TC, mg/dL	196 (168, 225)	
LDL, mg/dL	110 (85, 134)	
HDL, mg/dL	56.2 (46.1, 68.4)	
TG, mg/dL	93 (65, 136)	
hs-CRP, mg/dL	0.14 (0.05, 0.53)	
D-dimer, µg/mL	1.4 (0.7, 3.1)	
NIHSS at admission	5 (2, 16)	
Pre-stroke mRS	0 (0, 3)	

All values except for categorical data are represented as median (interquartile range)

*Abbreviations*: *ALT* alanine aminotransferase, *AST* aspartate aminotransferase, *BW* body weight, *HDL* high-density lipoprotein cholesterol, *hs-CRP* high-sensitivity C-reactive protein, *DVT* deep vein thrombosis, *LDL* low-density lipoprotein cholesterol, *mRS* modified Rankin scale score, *NGSP* National Glycohemoglobin Standardization Program, *N* number, *NIHSS* National Institutes of Health Stroke Scale, *TC* total cholesterol, *TG* triglyceride

**Table 2:** Comparison of variables between the two groups

	Patients with DVT	Patients without DVT	Chi-square value	*P*-value
N	161	756		
Ischemic stroke, n (%)	132 (82.0 %)	602 (79.6 %)	0.5	0.4924
Female sex, n (%)	106 (65.8 %)	343 (45.4 %)	22.4	< 0.0001
Age, years	82 (77, 89)	79 (71, 86)	21.0	< 0.0001
BMI, kg/m^2^	21.5 (19.1, 23.7)	22.1 (19.6, 24.5)	3.6	0.0569
BW, kg	52 (44, 60)	56 (47, 65)	11.5	0.0007
Hb, g/dL	12.6 (11.5, 13.8)	13.4 (12.3, 13.9)	21.3	< 0.0001
Plt, /µL	20.5 (17.1, 25.5)	20.9(17.2, 25.7)	0.2	0.6772
Alb, mg/dL	3.7 (3.4, 4.1)	4.0 (3.7, 4.2)	25.7	< 0.0001
AST, U/L	22 (19, 31)	23 (19, 28)	0.0	0.9744
ALT, U/L	15 (12, 21)	17 (12, 23)	6.3	0.0123
AST/ALT ratio	1.53 (1.22, 1.87)	1.38 (1.13, 1.73)	10.7	0.0011
Glucose, mg/dL	122 (106, 149)	123 (105, 153)	0.4	0.5314
HbA1c, % (NGSP)	5.8 (5.5, 6.3)	5.8 (5.5, 6.4)	0.2	0.6810
TC, mg/dL	194 (159, 226)	197 (170, 224)	0.3	0.5873
LDL, mg/dL	110 (82, 131)	111 (87, 135)	1.2	0.2794
HDL, mgl/dL	56.7 (44.3, 71.7)	55.8 (46.2, 68.2)	0.1	0.7125
TG, mg/dL	82 (61, 123)	95 (66, 141)	4.8	0.0279
hs-CRP, mg/dL	0.17 (0.07, 0.77)	0.13 (0.05, 0.47)	6.6	0.0103
D-dimer, µg/mL	2.7 (1.3, 6.0)	1.2 (0.6, 2.6)	60.2	< 0.0001
NIHSS at admission	8 (3,18)	5 (2,15)	12.3	0.0004
Pre-stroke mRS	2 (0, 3.5)	0 (0, 3)	16.1	< 0.0001

All values except for categorical data are represented as median (interquartile range)

*Alb* albumin, *ALT* alanine aminotransferase, *AST* aspartate aminotransferase, *BMI* body mass index, *BW* body weight, *Hb* hemoglobin, *HDL* high-density lipoprotein cholesterol, *hs-CRP* high-sensitivity C-reactive protein, *DVT* deep vein thrombosis, *LDL* low-density lipoprotein cholesterol, *mRS* modified Rankin scale score, *N* number, *NGSP* National Glycohemoglobin Standardization Program, *NIHSS* National Institutes of Health Stroke Scale, *P* probability, *Plt* platelet, *TC* total cholesterol, *TG* triglyceride

**Table 3:** Multiple logistic regression for deep vein thrombosis presence at the admission of stroke using receiver operating characteristics curves

	Odds ratio	*P*-value		AUC	BIC
			< 0.0001	0.687	774
Sex	2.04 (1.40–3.00)		0.0002		
D-dimer	1.05 (1.02–1.08)		0.0003		
TG	1.00 (0.99–1.00)		0.0560		
hs-CRP	1.08 (0.99–1.17)		0.0680		
ALT	0.99 (0.98–1.00)		0.2479		
NIHSS	1.01 (0.99–1.03)		0.2698		

*ALT* alanine aminotransferase, *AUC* area under the curve, *BIC* Bayesian information criterion, *hs-CRP* high-sensitivity C-reactive protein, *NIHSS* National Institutes of Health Stroke Scale at admission, *P* probability, *TG* triglyceride

**Table 4:** Threshold values for DVT presence using receiver operating characteristics curves from logistic regression analysis

	Sens (%)	Spec (%)	Odds ratio	*P*-value	AUC	BIC
Sex (1 vs. < 1)	65.8	54.5	2.31 (1.62–3.31)	< 0.0001	0.602	843
D-dimer (≥ 1.52 vs. < 1.52) μg/mL	70.0	59.6	1.06 (1.03–1.09)	< 0.0001	0.695	836

*AUC* area under the curve, *BIC* Bayesian information criterion, *DVT* deep vein thrombosis, *P* probability, *Sens* sensitivity, *Spec* specificity, *TG* triglyceride

**Table 5:** Cross tabulation table between patients with significant factors, DVT, Odds, and Odds ratio

	DVT presence	DVT absence	Odds	Odds ratio
N	161	756		
Patients with the two significant factors	78	154	0.5065	6.29
Patients with one significant factor	62	341	0.1818	2.26
Patients without any significant factors	21	261	0.0805	

The two significant factors are female sex and D-dimer ≥ 1.52 µg/mL

*DVT* deep vein thrombosis, *N* number

## Discussion

Our results demonstrated that DVT was present in 17.6 % of acute stroke patients at admission, and female sex and a high D-dimer level were significant factors indicating the presence of DVT at admission. The odds ratio of the presence of DVT in patients with both significant factors was 6.29. Therefore, in patients with both these significant factors at admission, IPC should be avoided or wl-US should be performed before IPC.

wl-US has been shown to have high sensitivity (94.0 %) and specificity (97.3 %) for detecting DVT [7]. However, wl-US is not always performed for stroke patients in most facilities. In contrast, D-dimer levels can be measured easily in any institution. A cut-off value of 0.5 µg/mL for D-dimer levels showed a sensitivity of 82.9 % and specificity of 32.7 % for detecting DVT in patients during hospitalization [6]. Furthermore, our results demonstrated that D-dimer was a significant factor for the presence of DVT at stroke admission. Therefore, D-dimer levels should be examined routinely at stroke admission.

According to previous studies, DVT was found on day 3 after stroke onset in 8.0 % of acute stroke patients and within 7 days of stroke onset in 8.7 % of acute stroke patients in Polish institutions [4, 5]. In comparison, the DVT frequency was high in our study at 17.5 %; this may be because our patients were older than those in previous studies, and US was performed within 72 h of arrival in our patients, compared to the performance of US within 7 days of stroke onset in a previous study [4, 5]. Female sex, elevated CRP levels, and pre-stroke disability were risk factors for DVT within 7 days of stroke onset, and elevated CRP and pre-stroke disability were independent risk factors for the presence of DVT [5]. However, D-dimer was not examined in the previous study [5]. Elevated CRP levels and pre-stroke disability were also found to be significant factors for the presence of DVT in our patients (Table 2). However, they were not independent because of multicollinearity.

The incidence of DVT has been reported to be approximately 50 % within 2 weeks in the absence of heparin prophylaxis in patients with acute hemiplegic stroke [13]. Patients with proximal subclinical DVT had a 15 % risk of fatal pulmonary embolism [13, 14]. Untreated below-knee DVT is associated with a 20 % risk of proximal extension [13], and the pulmonary embolism rate is reportedly 6.1 % in trauma patients with below-knee DVT [15]. On admission to the stroke rehabilitation unit, the prevalence of DVT in patients with stroke ranges from 12 to 40 % [16]. Therefore, the onset of DVT during hospitalization in primary stroke centers must be prevented. When DVT is not detected at admission, IPC can be used safely. If DVT is detected at admission in patients with ischemic stroke, anticoagulants may be started soon. The disuse of IPC may not induce thrombi fragmentation in patients with DVT at admission. Early anticoagulant therapy may protect against thromboembolism caused by DVT present at admission, and early IPC or anticoagulant therapy may prevent DVT development after admission. Overall, the frequency of symptomatic or critical DVT may decrease during hospitalization in primary stroke centers. Plasma D-dimer levels must be measured at the time of stroke, and patients with both significant factors, i.e., female sex and a D-dimer level ≥ 1.52 µg/mL, should immediately undergo wl-US, if possible.

### Limitations

Our study had several limitations. First, the sample size was small, and the study had a retrospective, cross-sectional design. Second, although US has a sensitivity of 94 % for detecting DVT, it cannot always detect DVT. Third, because most of the patients were Japanese, generalization of the study outcomes to non-Japanese populations may not be possible. There might be racial differences in the association between DVT-related factors and threshold values of factors associated with the presence of DVT. A prospective study including US and plasma D-dimer examination is required to determine the frequency of DVT at stroke admission and significant associated factors.

## Conclusions

The frequency of DVT at admission in acute stroke patients was high at 17.6 % in our institution. Female sex and high D-dimer levels were significant factors for the presence of DVT. Therefore, in patients with these two significant factors at admission, IPC should be avoided or wl-US should be performed before IPC.

## Supplementary Information

**Additional file 1.**

## Data Availability

The datasets analyzed during the current study are available from the corresponding author on reasonable request.

## Abbreviations

Alb: Albumin
ALT: Alanine aminotransferase
AST: Aspartate aminotransferase
DVT: Deep vein thrombosis
hs-CRP: High-sensitivity C-reactive protein
IPC: Intermittent pneumatic compression
mRS: Modified Rankin scale
NIHSS: National Institutes of Health Stroke Scale
US: Ultrasonography
VT: Venous thromboembolism
wl-US: Whole-leg ultrasonography
