# Supplementary material for: Frequency of deep vein thrombosis at admission for acute stroke and associated factors: a cross-sectional study
Source: Thromb J. 2021 Sep 6;19:62. doi: 10.1186/s12959-021-00315-5 (PMC8419953; doi:10.1186/s12959-021-00315-5)
Supplement: Supplementary file 1 — Additional file 1. [file 12959_2021_315_MOESM1_ESM.pdf]

Additional file 1. Spearman rank correlation coefficients between significant variables

r_s	Age	Sex	BW	Dd	Hb	Alb	TG	ALT	A/A ratio	CRP	NIH	mRS
Age		0.3	-0.5	0.5	-0.5	-0.3	-0.2	-0.3	0.4	0.1	0.3	0.5
Sex	0.3		-0.6	0.1	-0.3	-0.1	-0.1	-0.2	0.2	0.0	0.1	0.2
BW	-0.5	-0.6		-0.3	0.4	0.3	0.3	0.2	-0.4	-0.0	-0.2	-0.3
Dd	0.5	0.1	-0.3		-0.4	-0.5	-0.2	-0.2	0.3	0.3	0.3	0.3
Hb	-0.5	-0.3	0.4	-0.4		0.5	0.2	0.4	-0.3	-0.2	-0.2	-0.3
Alb	-0.3	-0.1	0.3	-0.5	0.5		0.1	0.2	-0.3	-0.3	-0.3	-0.4
TG	-0.2	-0.1	0.3	-0.2	0.2	0.1		0.0	-0.2	-0.0	-0.2	-0.2
ALT	-0.3	-0.2	0.2	-0.2	0.4	0.2	0.0		-0.6	-0.1	-0.0	-0.3
A/A ratio	0.4	0.2	-0.4	0.3	-0.3	-0.3	-0.2	-0.6		0.1	0.2	0.3
CRP	0.1	0.0	-0.0	0.3	-0.2	-0.3	-0.0	-0.1	0.1		0.2	0.2
NIH	0.3	0.1	-0.2	0.3	-0.2	-0.3	-0.2	-0.0	0.2	0.2		0.3
mRS	0.5	0.2	-0.3	0.3	-0.3	-0.4	-0.2	-0.3	0.3	0.2	0.3	

Alb, albumin; ALT, alanine aminotransferase; AST, aspartate aminotransferase; A/A ratio, AST/ALT ratio; BW, body weight; CRP, high-sensitivity C-reactive protein; Dd, D-dimer; Hb, hemoglobin; mRS, pre-stroke modified Rankin scale score; NIH, National Institutes of Health Stroke Scale at admission; $r_{s,}$ Spearman's rank correlation coefficient; TG, triglycerides
